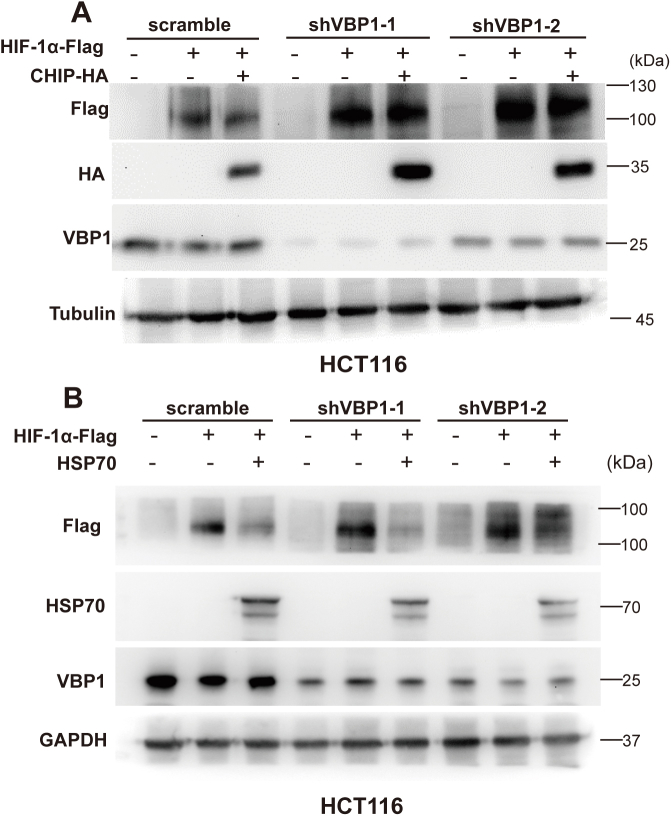# VBP1 negatively regulates CHIP and selectively inhibits the activity of hypoxia-inducible factor (HIF)-1α but not HIF-2α

**DOI:** 10.1016/j.jbc.2023.104829

**Published:** 2023-05-16

**Authors:** Yiming Yue, Yanfei Tang, Hao Huang, Dongdong Zheng, Cong Liu, Haifeng Zhang, Yunzhang Liu, Yun Li, Xiangrong Sun, Ling Lu

**Affiliations:** 1 Key Laboratory of Marine Drugs, The Ministry of Education of China, School of Medicine and Pharmacy, Ocean University of China, Qingdao, China; 2 Laboratory for Marine Drugs and Biological Products, Qingdao National Laboratory for Marine Science and Technology, Qingdao, China; 3 Department of Physiology and Pathophysiology, School of Basic Medicine, Qingdao University, Qingdao, China

**Keywords:** VBP1, CHIP, pVHL, HIF-1α, HIF-2α

## Abstract

Hypoxia-inducible factor-1 (HIF-1) is a critical transcription factor that regulates the expression of genes involved in cellular adaptation to low oxygen levels. Aberrant regulation of the HIF-1 signaling pathway is linked to various human diseases. Previous studies have established that HIF-1α is rapidly degraded in a von Hippel-Lindau protein (pVHL)-dependent manner under normoxic conditions. In this study, we find that pVHL binding protein 1 (VBP1) is a negative regulator of HIF-1α but not HIF-2α using zebrafish as an *in vivo* model and *in vitro* cell culture models. Deletion of *vbp1* in zebrafish caused Hif-1α accumulation and upregulation of Hif target genes. Moreover, *vbp1* was involved in the induction of hematopoietic stem cells (HSCs) under hypoxic conditions. However, VBP1 interacted with and promoted the degradation of HIF-1α in a pVHL-independent manner. Mechanistically, we identify the ubiquitin ligase CHIP and HSP70 as new VBP1 binding partners and demonstrate that VBP1 negatively regulated CHIP and facilitated CHIP-mediated degradation of HIF-1α. In patients with clear cell renal cell carcinoma (ccRCC), lower VBP1 expression was associated with worse survival outcomes. In conclusion, our results link VBP1 with CHIP stability and provide insights into underlying molecular mechanisms of HIF-1α-driven pathological processes.

Multicellular eukaryotic organisms depend on oxygen for normal development and physiological functions (1). Several human diseases including cancer, stroke, anemia, and others are associated with low oxygen levels or hypoxia (2). Hypoxia-inducible factors (HIFs) are key sensors of oxygen availability that mediate cell survival and adaptive mechanisms and processes related to low oxygen levels (3). HIF proteins are evolutionarily conserved transcriptional factors with an oxygen-sensitive α subunit and a constitutive β subunit. As the major paralogues, HIF-1α and HIF-2α have unique and overlapping functions in response to hypoxia (4, 5, 6, 7). For example, both HIF-1α and HIF-2α regulate tumor progression by directly modulating the expression of several unique and shared target genes, but multiple studies have shown opposing effects of these two HIF-α proteins on critical tumor-related factors such as c-Myc, p53, and mTORC1 (8, 9, 10, 11). Direct or indirect targeting of HIF-1α and/or HIF-2α is an important therapeutic strategy for patients with anemia and different types of cancers (12, 13). Therefore, unraveling the regulatory mechanisms of HIF-1α and HIF-2α are essential for developing novel therapies for human diseases.

Several major mechanisms underlying the regulation of HIF-α stability during normoxia and hypoxia have been delineated. HIF-α is mainly degraded *via* a pVHL-dependent mechanism (14, 15, 16, 17). In normoxic conditions, HIF-α proteins undergo hydroxylation by the prolyl hydroxylase domain proteins (PHDs) followed by polyubiquitination by the E3 ubiquitin ligase, von Hippel-Lindau protein (pVHL); subsequently polyubiquitinated HIF-α proteins are targeted for proteasomal degradation. However, hydroxylation of HIF-α is inhibited under hypoxic conditions. Therefore, the stabilized HIF-α proteins translocate to the nucleus, dimerize with HIF-β, and the HIF-α/HIF-β complex induces the expression of the HIF-target genes. HIF-α stability is also regulated by pVHL-independent pathways. For example, HIF-α proteins undergo pVHL-independent ubiquitination and proteasomal degradation through E3 ubiquitin-protein ligases such as MDM2, RACK1, and HUWE1 (18, 19, 20). Furthermore, lysosomal degradation of HIF-1α is mediated *via* heat shock protein (HSP70)-related autophagy (21, 22).

The von Hippel-Lindau binding protein (VBP1) or prefoldin three is a subunit of the prefoldin complex, which protects pVHL against aggregation and degradation (23). Prefoldin consists of six subunits including two α-subunits (PFD3 and PFD5) and four β-subunits (PFD1, PFD2, PFD4, and PFD6) and is involved in the proper folding of newly synthesized proteins (24). Eukaryotic prefoldin shows higher substrate specificity than prokaryotic prefoldin. VBP1 participates in the correct folding of tubulin and actin (25). In *Drosophila*, deletion of VBP1 causes loss of meiotic spindle integrity (25). Loss of VBP1 is lethal in *Caenorhabditis elegans* because of a high tubulin requirement in the mitotic cells of the embryo (26). In addition to the co-chaperone role in protein folding, VBP1 also triggers polyubiquitylation and proteasomal degradation of integrase (27). Furthermore, VBP1 facilitates proteasomal and autophagy-mediated degradation of hMSH4 (28). We recently reported that VBP1 modulates Wnt/β-catenin signaling by mediating the stability of TCF/LEFs (29). A recent study reported that VBP1 facilitates HIF-1α degradation by stabilizing pVHL (30). However, whether VBP1 modulates HIF signaling pathway *in vivo* and the underlying mechanism has not been established.

In the present study, we identified VBP1 as a HIF-1α-interacting protein. VBP1 selectively regulates the degradation of HIF-1α but not HIF-2α in a pVHL-independent manner. We presented evidence that VBP1 modulates the HIF signaling pathway *in vivo* with a HIF reporter transgenic zebrafish line. Importantly, we further identified that CHIP acts as a critical regulator to control VBP1-mediated HIF-1α degradation. Thus, our data provided new mechanistic insights into HIF-1α regulation.

## Results

### Hif signaling pathway is negatively regulated by Vbp1 in zebrafish

**Figure 1:**
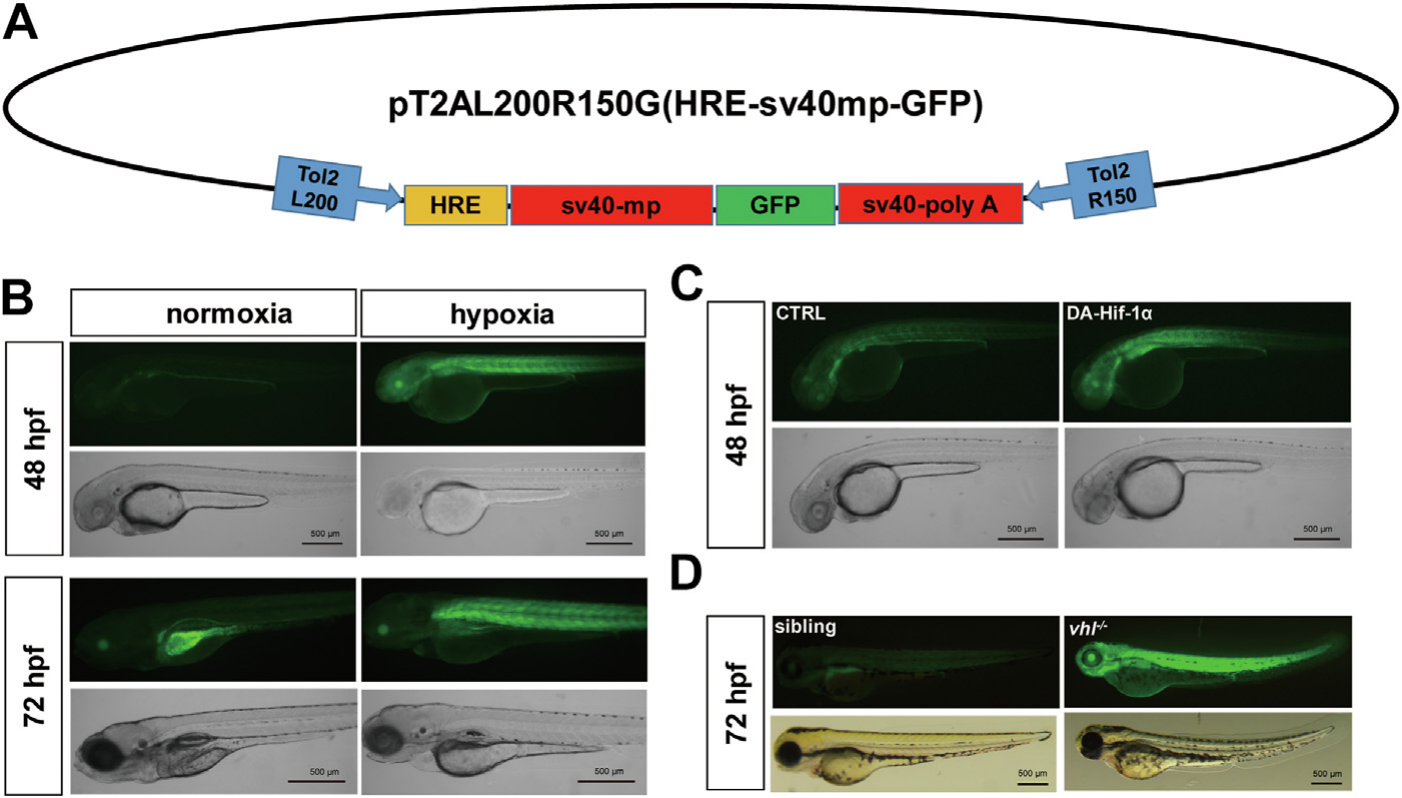
**Generation of the transgenic *HRE-sv40mp:GFP* reporter zebrafish.***A*, Schematic representation of the Tol-2 vector used to generate the *Tg (hre-sv40mp: GFP)* zebrafish. The construct includes a 68-bp fragment from the hypoxia-response element (HRE) of the human ENO1 gene and the SV40 minimal promoter (SV40mp). *B*, Representative images show the *Tg (hre-sv40mp:GFP)* embryos at 48 and 72 hpf under normoxic or hypoxic conditions. *Tg (hre-sv40mp:GFP)* embryos were exposed to hypoxia (10% O_2_) for 24 or 48 h from 24 hpf. Transgenic embryos show strong GFP expression during hypoxia. However, GFP expression is observed only in the visceral organs in the transgenic embryos exposed to normoxia. *C*, *Tg(hre-sv40mp:GFP)* embryos injected with the dominant-active (DA) HIF-1α mRNA at the 1-cell stage show strong GFP expression at 48 hpf. *D*, Transgenic zebrafish in the *vhl*^*−/−*^ background show strong GFP fluorescence compared to the sibling embryos at 72 hpf. Scale bar, 500 μm.

**Supporting Figure S1:**
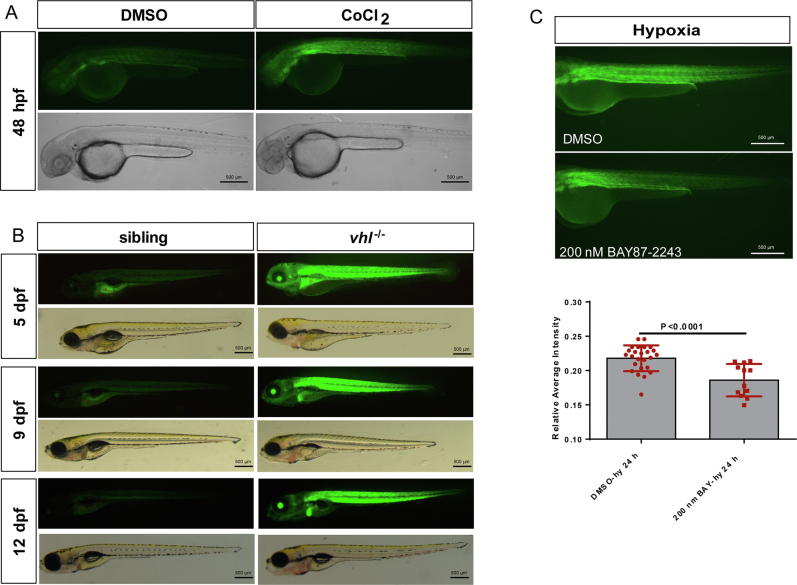


To determine the regulatory role of VBP1 in the HIF signaling pathway *in vivo*, we generated the transgenic HIF reporter zebrafish expressing GFP driven by the hypoxia-responsive element (HRE) of the human *ENO1* gene binding sites, named *Tg(hre-sv40mp:GFP)* (Fig. 1*A*). In normoxia, there was no obvious fluorescence before 24 h post-fertilization (hpf). GFP expression was detected in the visceral organs at 48 hpf, possibly suggesting endogenous HIF activity. To verify if *Tg(hre-sv40mp:GFP)* embryos could act as a live reporter of hypoxia, we exposed the transgenic embryos to the hypoxic environment (10% O_2_) for 24 or 48 h from 24 hpf. The results showed that strong GFP fluorescence expression was triggered in *Tg(hre-sv40mp:GFP)* embryos (Fig. 1*B*). Strong GFP fluorescence was observed mainly in the somite of transgenic zebrafish embryos exposed to hypoxia, thereby indicating significant activity of HIF in this region. Transgenic embryos treated with CoCl_2_, a chemical inducer of HIF-1, showed strong GFP expression (Fig. S1*A*). To further confirm that the reporter activities were dependent on the HIF transcription factor, we synthesized *in vitro* human mRNA encoding dominant active (DA) HIF-1α, in which the conserved proline hydroxylation sites (P402A/P564A) were mutated. The injection of DA HIF-1α mRNAs into 1-cell stage *Tg(hre-sv40mp:GFP)* embryos showed strong GFP expression at 24 hpf; GFP fluorescence persisted in the somite for at least 48 h (Fig. 1*C*). Activation of the *hre-sv40mp:GFP* transgene was also analyzed in the *vhl* mutant allele background wherein two nucleotides of *vhl* were deleted (31). GFP expression was obviously higher in the *vhl* homozygous mutant zebrafish embryos (*vhl*^*−/−*^) compared with the sibling embryos (Figs. 1*D* and S1*B*). Furthermore, treatment with BAY 87-2243, a pan HIF-1 inhibitor, significantly reduced GFP fluorescence compared with the DMSO control group (Fig. S1*C*). Overall, these data suggested that *Tg(hre-sv40mp:GFP)* transgenic fish expressed GFP in a manner that was dependent on Hif signaling.

**Figure 2:**
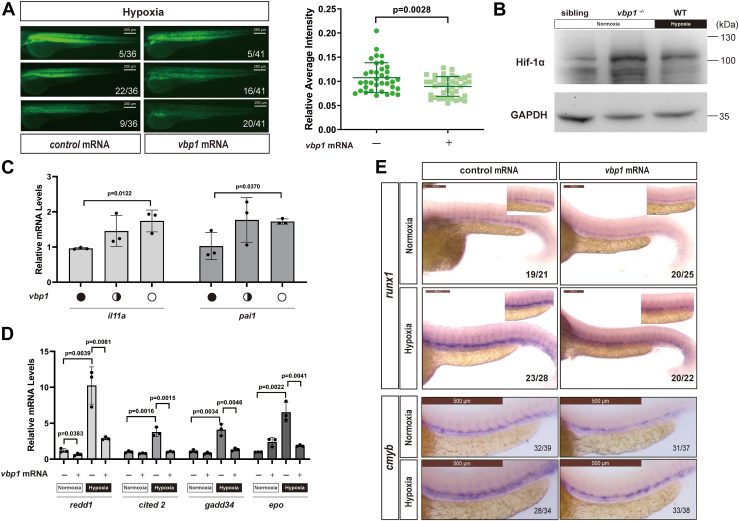
**Vbp1 negatively regulates the HIF-1α signaling pathway in zebrafish.***A*, Representative images show GFP fluorescence in the *Tg (hre-sv40mp:GFP)* embryos that were injected with mCherry or *vbp1* mRNA at the 1-cell stage and exposed to hypoxia for 48 h from 24 hpf. The histograms show average GFP fluorescence intensity in the somite of the *Tg(hre-sv40mp:GFP)* larvae (n ≥ 36) at 72 hpf. *B*, Western blot shows Hif-1α protein levels in the sibling, *vbp1*^*−/−*^*,* wt (hypoxia treatment) larvae at four dpf. As shown, Hif-1α is upregulated in the *vbp1* mutants. *C*, The relative mRNA levels of hypoxia-responsive genes, namely, *il11a*, and *pai1* in the WT, *vbp1*^*+/−*^, and *vbp1*^*−/−*^ larvae. The data show upregulation of these hypoxia-responsive genes in the *vbp1* mutant zebrafish (n = 3, biologically independent extracts). *D*, Effect of forced expression of *vbp1* on *redd1*, *cited2*, *gadd34*, and *epo* mRNA expression. Embryos injected with GFP or *vbp1* capped mRNA was raised to 24 hpf and then exposed to hypoxia (10% O_2_) for 24 h. The mRNA levels of the indicated genes were determined by qPCR. (n = 3, biologically independent extracts). *E*, Representative images show both *runx1* and *cmyb* expression in the zebrafish embryos injected with control or *vbp1* mRNAs under normoxic and hypoxic conditions. As shown, *runx1*/*cmyb* expression is reduced in the *vbp1*-injected embryos under hypoxia conditions. The brightfield images of WISH for *runx1*/*cmyb* expression are shown in the WT embryos at 36 hpf under normoxia and hypoxia exposure for 8 h starting at 28 hpf (lateral views). Scale bar, 250 μm and 500 μm representatively. All data are represented as means ± SD. n/n, number of embryos showing representative phenotype/total number of embryos examined. HIF-1, Hypoxia-inducible factor-1; pVHL, von Hippel-Lindau protein; VBP1, pVHL binding protein 1.

We then microinjected synthesized zebrafish *vbp1* mRNA into *Tg(hre-sv40mp:GFP)* embryos at the one-cell stage. Exposure of the transgenic embryos at 24 hpf to hypoxia (10% O_2_) for 48 h induced strong GFP fluorescence, but the expression of GFP was reduced in the transgenic embryos injected with the zebrafish *vbp1* mRNA under hypoxic conditions (Fig. 2*A*). We then analyzed Hif-1α protein levels and expression levels of Hif-1α target genes in the control and *vbp1*^*−/−*^ *embryos* to confirm the role of Vbp1. The levels of Hif-1α protein were higher in the *vbp1* homozygous null zebrafish embryos compared to the corresponding controls (Fig. 2*B*). As a positive control, we also found that hypoxia treatment led to the increase of Hif-1α protein (Fig. 2*B*). Moreover, the mRNA levels of *il11a* and *pai1*, two HIF-1 target genes, increased in the *vbp1* homozygous null zebrafish embryos than the corresponding controls (Fig. 2*C*). We cannot detect the levels of zebrafish Hif-2α protein due to a lack of appropriate antibodies. Likewise, forced expression of Vbp1 resulted in significant decreases in mRNA levels of several well-known HIF-1 target genes including *redd1*, *cited2*, *gadd34* and *epo* after hypoxia treatment (Fig. 2*D*). These data confirmed that Vbp1 negatively regulated Hif signaling activity.

HIF-1α was recently shown to have an important role in the formation of hematopoietic stem cells (HSCs) in zebrafish and other vertebrates (32). In zebrafish, a specific subset of endothelial cells from the ventral wall of the dorsal aorta (VDA) develops into HSCs *via* endothelial-to-hematopoietic transition (EHT). Hypoxia conditions strongly stimulate HSC formation, and HSC marker gene expression, such as *runx1, cmyb,* decreases in *hif-1* mutant zebrafish (32). Therefore, we investigated if Vbp1 regulated the formation of HSCs during hypoxia by microinjecting zebrafish embryos with *vbp1* mRNA and subsequently exposing them to hypoxia for 8 h. Then, we analyzed *cmyb/runx1* expression levels using the whole-mount RNA in-situ hybridization (WISH) assay. In the embryos exposed to hypoxia, expression of both *runx1* and *cmyb* was higher at 36 hpf in the VDA compared with the embryos exposed to normoxia, but their expression was lower in the *vbp1*-injected embryos during hypoxia (Fig. 2*E*). These data suggested that Vbp1 impacts the HSC induction *in vivo*.

### VBP1 acts as a negative regulator of HIF-1α but not HIF-2α

**Figure 3:**
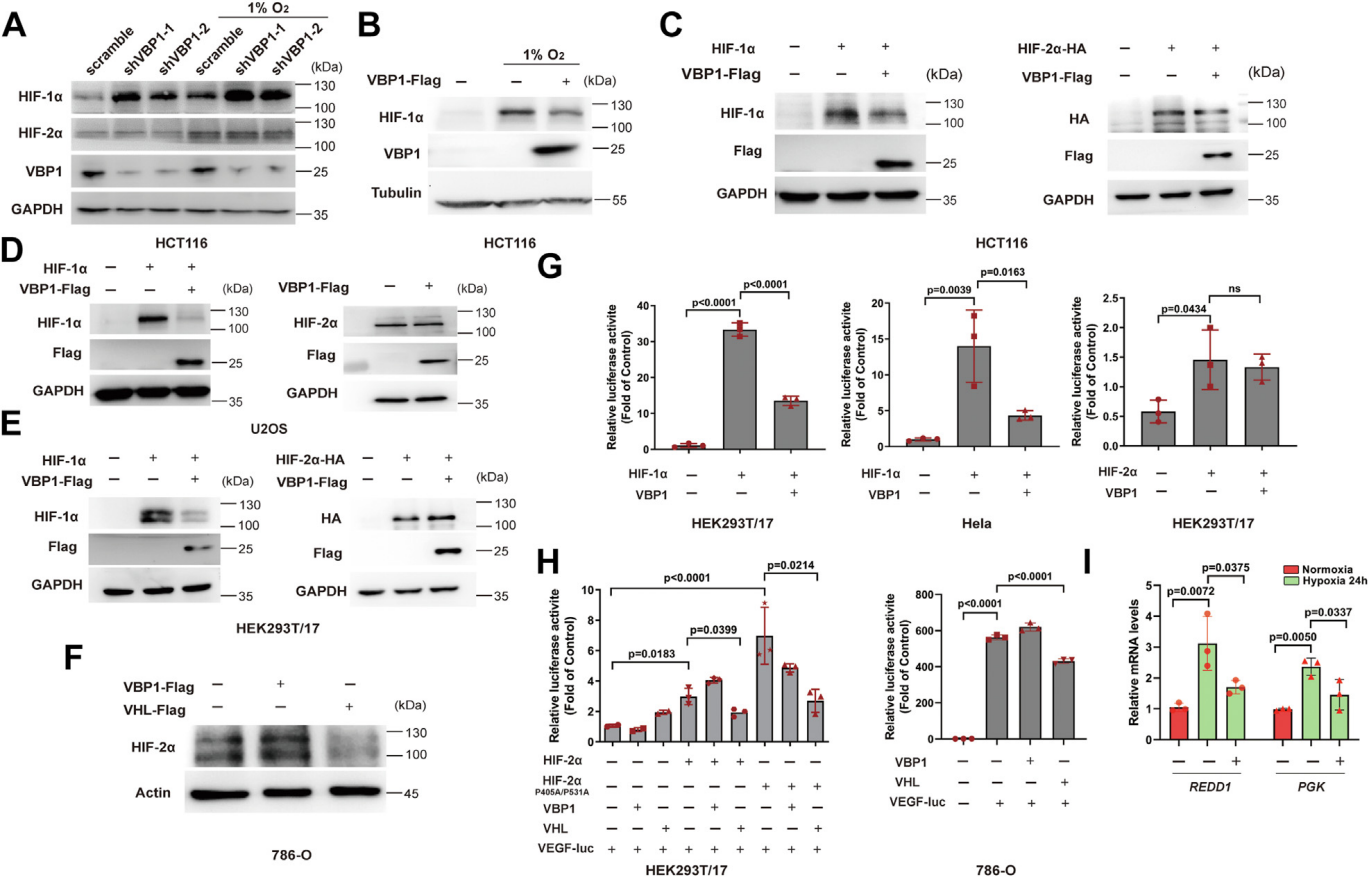
**VBP1 negatively regulates HIF-1α but not HIF-2α.***A*, Western blot shows protein levels of HIF-1α but not HIF-2α were elevated in VBP1 knockdown HCT116 cells. The cells were stably transfected with the lentiviral pLKO vector containing specific shRNAs targeting VBP1 or control scrambled shRNA and exposed to either 21% or 1% O_2_ for 6 h. *B*, HIF-1α protein levels were downregulated under hypoxic conditions. Western blotting analysis of control and VBP1-overexpressing HCT116 cells cultured at the indicated oxygen levels for 24 h. *C*–*E*, Overexpression of VBP1 decreased the HIF-1α but not HIF-2α protein levels. HCT116, U2OS, and HEK293T/17 cells were transfected with the indicated plasmids, and the proteins were detected by Western blotting analysis. (−), the cells transfected with the empty vector control; (+), the cells transfected with the indicated vector. *F*, Overexpression of VHL decreased HIF-2α protein levels but VBP1 failed to decrease it. 786-O cells were transfected with the indicated plasmids, and the proteins were detected by Western blotting analysis. *G*, ENO1 promotor-luciferase reporter activity in the HEK293T/17 and the HeLa cells show that VBP1 decreases HIF-1α but not HIF-2α transcriptional activity. The firefly luciferase activities were normalized to the Renilla luciferase activity. n = 3 biological replicate samples. *H*, VEGF promotor-luciferase reporter activity in the HEK293T/17 and the 786-O cells show that VBP1 failed to decrease HIF-2α or HIF-2α (P405A/P531A) transcriptional activity. The firefly luciferase activities were normalized to the Renilla luciferase activity. n = 3 biological replicate samples. *I*, RT-qPCR analysis of HIF target gene induction in response to hypoxia in HEK293T/17 cells transfected with empty vector or Flag-tagged VBP1 vector. The data are represented as means ± SD. n = 3 biologically independent extracts. ns, not significant. HIF-1, Hypoxia-inducible factor-1; pVHL, von Hippel-Lindau protein; VBP1, pVHL binding protein 1.

A previous study showed that VBP1 modulates the stability of HIF-1α (30). Therefore, we investigated if VBP1 co-regulated HIF-2α protein stability and levels. The short hairpin RNA (shRNA)-mediated knockdown of VBP1 in HCT116 cells increased HIF-1α protein levels under both normoxic and hypoxic conditions but did not affect HIF-2α protein levels (Fig. 3*A*). Since HIF-1α protein levels are tightly controlled by oxygen availability, we investigated whether VBP1-dependent reduction of HIF-1α protein levels was sensitive to hypoxia. However, the effects of VBP1 on the HIF-1α protein levels were independent of the oxygen levels, and HIF-1α protein levels were downregulated efficiently under hypoxic conditions (Fig. 3*B*). Next, we examined the effects of VBP1 overexpression on the levels of HIF-1α and HIF-2α. The overexpression of VBP1 decreased HIF-1α protein levels but did not affect HIF-2α protein levels in the HCT116, U2OS, and HEK293T/17 cells (Fig. 3, *C*–*E*). To further determine whether VBP1 modulates endogenous HIF-2α protein levels, we used pVHL-deficient renal carcinoma (786-O) cells which have high endogenous levels of HIF-2α protein. Similarly, although ectopic expression of VHL decreased HIF-2α protein levels, the overexpression of VBP1 has no effect on endogenous HIF-2α protein levels in this VHL mutant cell line (Fig. 3*F*). Using a luciferase reporter construct, which contains an HRE from the human ENO1 gene upstream of SV40 promoter and FLuc coding sequences, we then assessed whether VBP1 affects the transcriptional activity of HIF-α. The luciferase reporter gene was significantly induced by HIF-1α overexpression. The relative luciferase activity was significantly reduced in the HEK293T/17 and HeLa cells co-transfected with the VBP1 and HIF-1α but was not affected in HEK293T/17 cells co-transfected with the VBP1 and HIF-2α (Fig. 3*G*). As ENO1 promoter-luciferase reporter is not ideal to measure HIF-2α transcriptional activity, we also use our previously constructed VEGF promoter-luciferase reporter plasmid to detect the effect of VBP1 on HIF-2α transcriptional activity (33). Although co-transfected with the VHL and HIF-2α decreased the luciferase activity, VBP1 did not show any effect (Fig. 3*H*). Furthermore, the expression of HIF-1α target genes, including REDD1 and PGK were significantly induced during hypoxia but were significantly decreased by VBP1 overexpression (Fig. 3*I*). These data demonstrated that VBP1 acted as a negative regulator of HIF-1α but not HIF-2α.

### VBP1 interacts with HIF-1α and induces HIF-1α degradation through proteasomal and autophagy pathways

**Figure 4:**
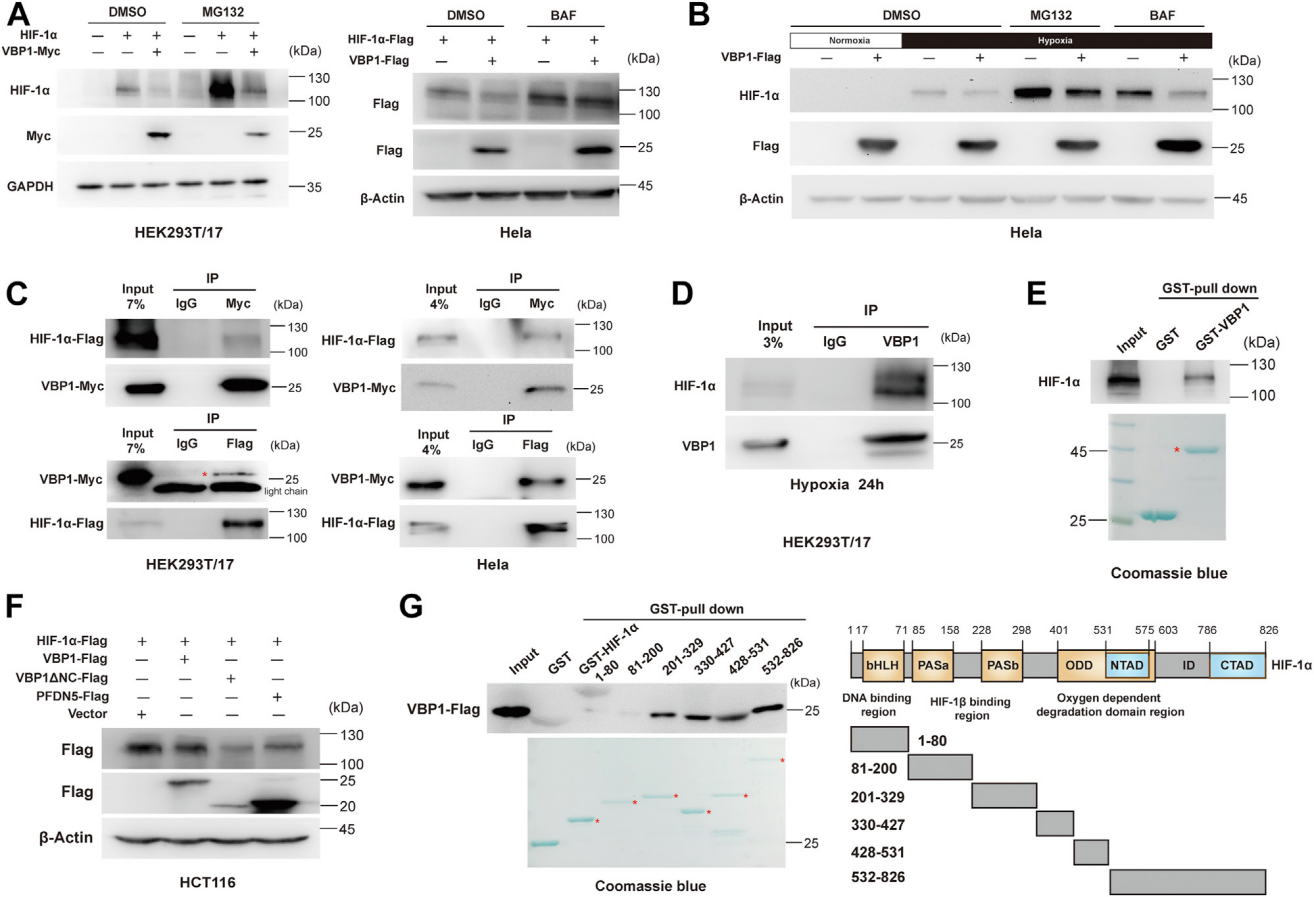
**VBP1 interacts with HIF-1α and induces HIF-1α degradation.***A*, The downregulation of HIF-1α protein caused by ectopic expression of VBP1 was blocked by MG132 and BAF. Cells were transfected with the indicated plasmids and harvested 24 h after transfection; MG132 (*left panel*) or BAF (*right panel*) were added to the culture medium at 16 h after transfection. and the proteins were detected by Western blotting analysis. (−), the cells transfected with the empty vector control; (+), the cells transfected with the indicated vector. *B*, The downregulation of endogenous HIF-1α protein caused by ectopic expression of VBP1 in the hypoxic cells could be rescued by treatment with MG132 and BAF. HeLa cells transfected with VBP1-Flag or a control plasmid were incubated under normoxia or hypoxia for 24 h, and treated with vehicle or 10 μM MG132 or 50 nM BAF for 8 h. Cell lysates were immunoblotted for HIF-1α. *C*, VBP1 interacts with HIF-1α. The interaction between VBP1-Myc and Flag-HIF-1α was analyzed in HEK293T/17 cells (*left panel*) and HeLa cells (*right panel*) by reciprocal Co-IP as indicated. *D*, Endogenous VBP1 interacts with HIF-1α in HEK293T/17 cells. Anti-VBP1 antibody was used for IP. *E*, GST pull-down assay results show the interaction between HIF-1α and GST-tagged VBP1. *F*, Overexpression of VBP1ΔNC resulted in a decrease of HIF-1α. HCT116 cells were transfected with the indicated plasmids and harvested after transfection of 24 h and the proteins were detected by Western blotting analysis. *G*, Mapping the interaction domain of HIF-1α with VBP1. GST pull-down assays were performed with GST or GST fusion proteins containing the indicated amino acid residues of HIF-1α (*top*) and the whole-cell lysates of HEK293T/17 cells expressing VBP1-Flag. bHLH, basic helix-loop-helix; HIF-1, Hypoxia-inducible factor-1; PAS, Per-ARNT-Sim; pVHL, von Hippel-Lindau protein; TAD, transactivation domain; VBP1, pVHL binding protein 1.

**Supporting Figure S2:**
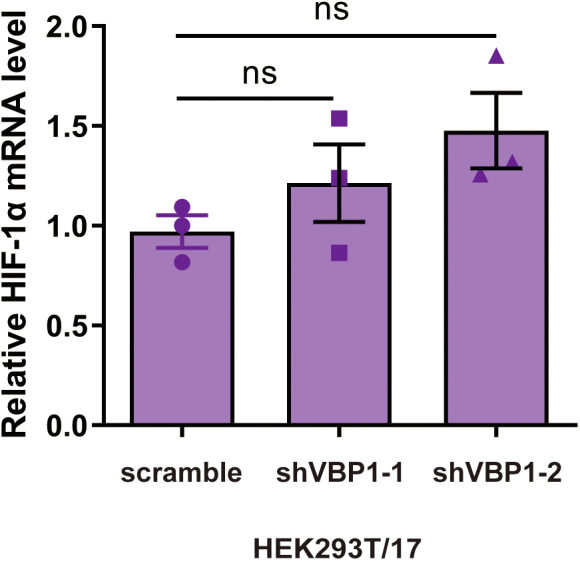


We then investigated the mechanism by which VBP1 negatively regulated HIF-1α. Changes in VBP1 levels did not alter the mRNA levels of HIF-1α (Fig. S2). We then examined whether VBP1 inhibited HIF-1α protein levels through degradation. As previously reported (30), VBP1 overexpression shortened the half-life of HIF-1α when treated with the translational inhibitor cycloheximide (CHX) (data not shown). This suggested that VBP1 did not regulate HIF-1α protein synthesis. We and others have shown that VBP1 induced ubiquitination of the HIF-1α protein in cultured cells (29, 30). Surprisingly, MG132 (a proteasomal inhibitor) treatment only partly rescued VBP1-mediated HIF-1α degradation (Fig. 4*A*). We then treated HeLa cells with BAF (lysosomal proteolysis inhibitor) and found that inhibition of autophagy also reduced VBP1-induced HIF-1α degradation (Fig. 4, *A* and *B*). These results suggested that HIF-1α underwent VBP1-mediated proteasomal-dependent and autophagy-dependent degradation. Furthermore, co-immunoprecipitation (Co-IP) assay results showed a physical association between HIF-1α and VBP1 in the HEK293T/17 cells and HeLa cells (Fig. 4*C*). Moreover, HIF-1α interacts with VBP1 endogenously (Fig. 4*D*). GST pull-down assays also demonstrated strong binding between HIF-1α and GST-VBP1 (Fig. 4*E*). We also examined the interaction between HIF-2α and VBP1 in HEK293T/17 cells co-transfected with plasmids expressing Flag-tagged VBP1 and HA-tagged HIF-2α, but no interaction was observed (data not shown).

Next, we analyzed if VBP1 altered HIF-1α stability and degradation through its chaperone function. We generated a truncated VBP1 (VBP1ΔNC), which alters the chaperone function of VBP1 (23). VBP1ΔNC overexpression decreased HIF-1α levels as efficiently as the full-length VBP1 (Fig. 4*F*). Prefoldin is a heterohexameric co-chaperone protein, PFDN3 (VBP1) and PFDN5 are the two α-like subunits, and all six subunits could stabilize nascent proteins (34). Interestingly, we found that PFDN5 overexpression also decreased HIF-1α protein levels (Fig. 4*F*). Overall, the above results suggested that VBP1 induced HIF-1α degradation in a folding-independent manner *via* proteasomal and autophagy-related pathways.

We then mapped the HIF-1α domain that interacts with VBP1 by generating the following GST-tagged HIF-1α truncations: (1) basic helix-loop-helix/PAS (bHLH/PAS) domain: GST-HIF-1α (1–80), GST-HIF-1α (81–200), and GST-HIF-1α (201–329); (2) oxygen-dependent degradation domain: GST-HIF-1α (330–427) and GST-HIF-1α (428–531); (3) and transactivation domain: GST-HIF-1α (532–826). GST pull-down assays demonstrated that VBP1 was strongly bound to GST-HIF-1α (201–329)，GST-HIF-1α (330–427), and associated with GST-HIF-1α (428–531) and GST-HIF-1α (532–826) (Fig. 4*G*). VBP1 might, therefore, potentially affect the degradation and transcriptional activity of HIF-1.

### VBP1 modulates HIF-1α protein levels in a pVHL-independent manner

**Figure 5:**
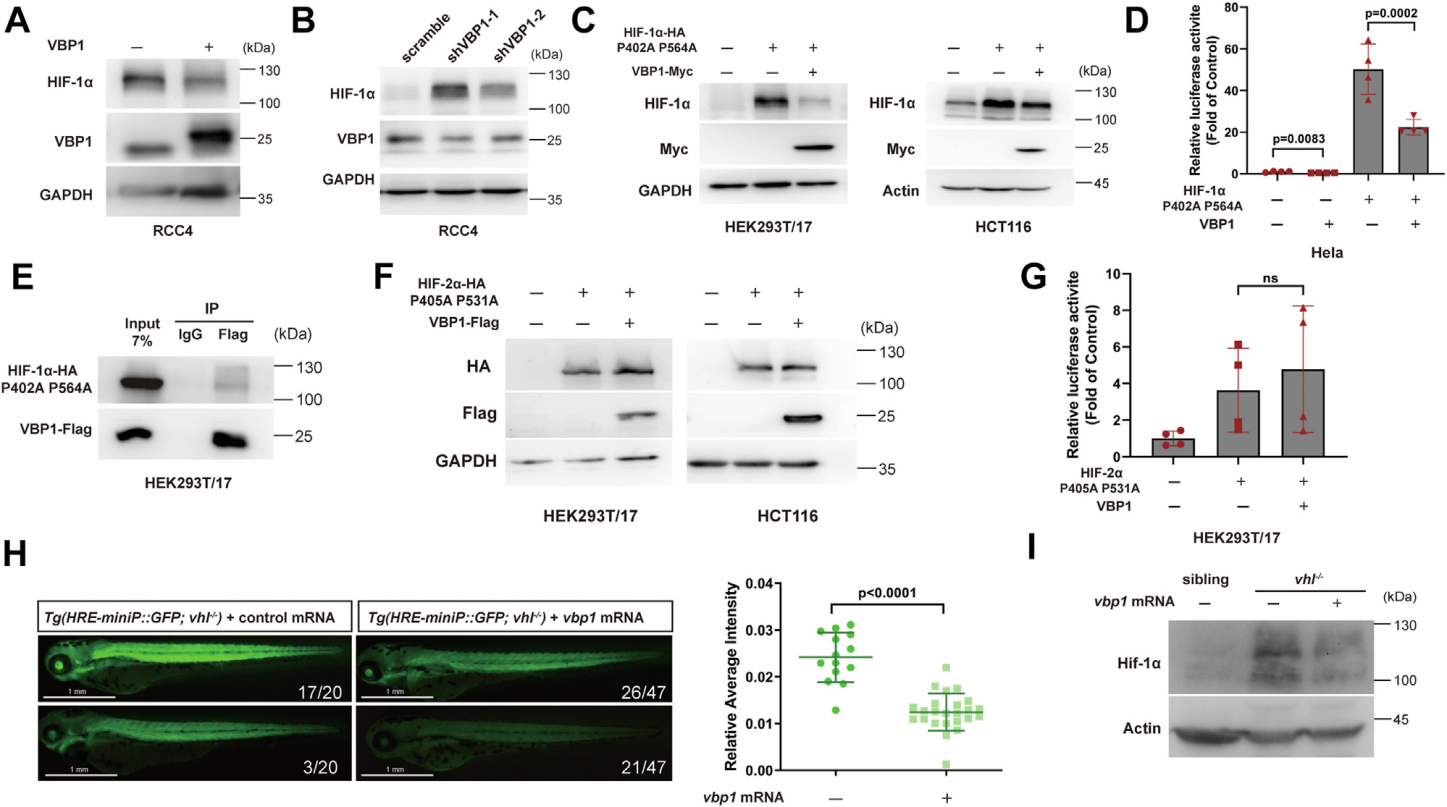
**VBP1 negatively modulates HIF-1α protein levels in a pVHL-independent manner.***A*, Overexpression of VBP1 decreased the HIF-1α protein levels in pVHL-deficient RCC4 cells. RCC4 cells were stably transfected with the control or pCS2-VBP1 plasmid, and the proteins were detected by Western blotting analysis. *B*, Protein levels of HIF-1α were elevated in VBP1 knockdown RCC4 cell lines. Immunoblot analysis of cells stably transfected with lentiviral pLKO plasmid with VBP1-specific shRNA or scrambled control shRNA. *C*, VBP1 decreases HIF-1α (P402A/P564A) protein levels. HEK293T/17 and HCT116 cells were transfected with the indicated plasmids, and the proteins were detected by Western blotting analysis. *D*, HIF luciferase reporter assay results in HeLa cells show that VBP1 decreased HIF-1α (P402A/P564A) transcriptional activity. Data are represented as means ± SD (n > 3 biological replicate samples). *E*, VBP1 interacts with HIF-1α (P402A/P564A). Co-IP was performed with lysates from HEK293T/17 cells co-transfected with plasmids overexpressing VBP1-Flag and HA-tagged HIF-1α (P402A/P564A). *F*, VBP1 has no effect on the protein levels of HIF-2α (P405A/P531A). Immunoblot analysis of cells transfected with the indicated plasmids. *G*, HIF luciferase reporter assay results in HEK293T/17 cells show that VBP1 does not affect the HIF-2α (P405A/P531A) transcriptional activity. ns, not significant. n > 3 biological replicate samples. *H*, Vbp1 negatively modulates the HIF-1α signaling pathway in *Tg (hre-sv40mp:GFP)* zebrafish in the *vhl*^*−/−*^ background. *Tg(hre-sv40mp:GFP)* embryos were injected with control mRNA or *vbp1* mRNA, and images were taken in 72 hpf. The histograms show the average values (means ± SD) of GFP intensity in the somite of the *Tg(hre-sv40mp:GFP) vhl*^*−/−*^ larvae at 72 hpf. n/n, number of embryos showing representative GFP fluorenes/total number of embryos examined. *I*, Vbp1 negatively modulates the Hif-1α protein levels in *vhl*^*−/−*^ zebrafish. Zebrafish embryos injected with control mRNA or *vbp1* mRNA were collected at 72 hpf and the proteins were detected by Western blotting analysis. HIF-1, Hypoxia-inducible factor-1; pVHL, von Hippel-Lindau protein; VBP1, pVHL binding protein 1.

Our results revealed that VBP1-induced HIF-1α protein degradation but did not affect HIF-2α protein stability and degradation. This suggested that VBP1 regulated HIF-1α protein degradation independent of pVHL. To test this hypothesis, we investigated whether VBP1 regulated HIF-1α in the pVHL-deficient RCC4 renal carcinoma cells, which demonstrate high endogenous levels of HIF-1α and HIF-2α proteins. HIF-1α protein levels were reduced in the VBP1-overexpressing RCC4 cells compared to the control RCC4 cells (Fig. 5*A*). VBP1 knockdown increased HIF-1α protein levels (Fig. 5*B*). Next, we analyzed if VBP1 regulated the degradation of a mutant HIF-1α protein, which contains two proline-to-alanine substitutions, P402A and P564A, and is resistant to pVHL-mediated degradation (16). VBP1 overexpression decreased the protein levels and transcriptional activity of HIF-1α (P402A/P564A) (Fig. 5, *C* and *D*). Co-IP assays demonstrated that the interaction between the mutant HIF-1α (P402A/P564A) and VBP1 was unaffected by the mutations (Fig. 5*E*). In contrast, VBP1 did not affect the protein levels and the transcriptional activity of the mutant HIF-2α (P405A/P531A) (Figs. 3*H* and 5, *F* and *G*). Finally, to confirm the independence of VBP1-mediated degradation of HIF-1α from the pVHL-mediated degradation pathway, we used the *Tg (hre-sv40mp:GFP)* zebrafish in the *vhl*^*−/−*^ background. GFP fluorescence and Hif-1α protein levels were significantly reduced in the *vhl*^*−/−*^ zebrafish microinjected with the zebrafish *vbp1* mRNA (Fig. 5, *H* and *I*). Taken together, these data provided strong evidence that VBP1 regulated HIF-1α levels independent of the pVHL-mediated degradation pathway.

### CHIP is required for VBP1-mediated degradation of HIF-1α

**Figure 6:**
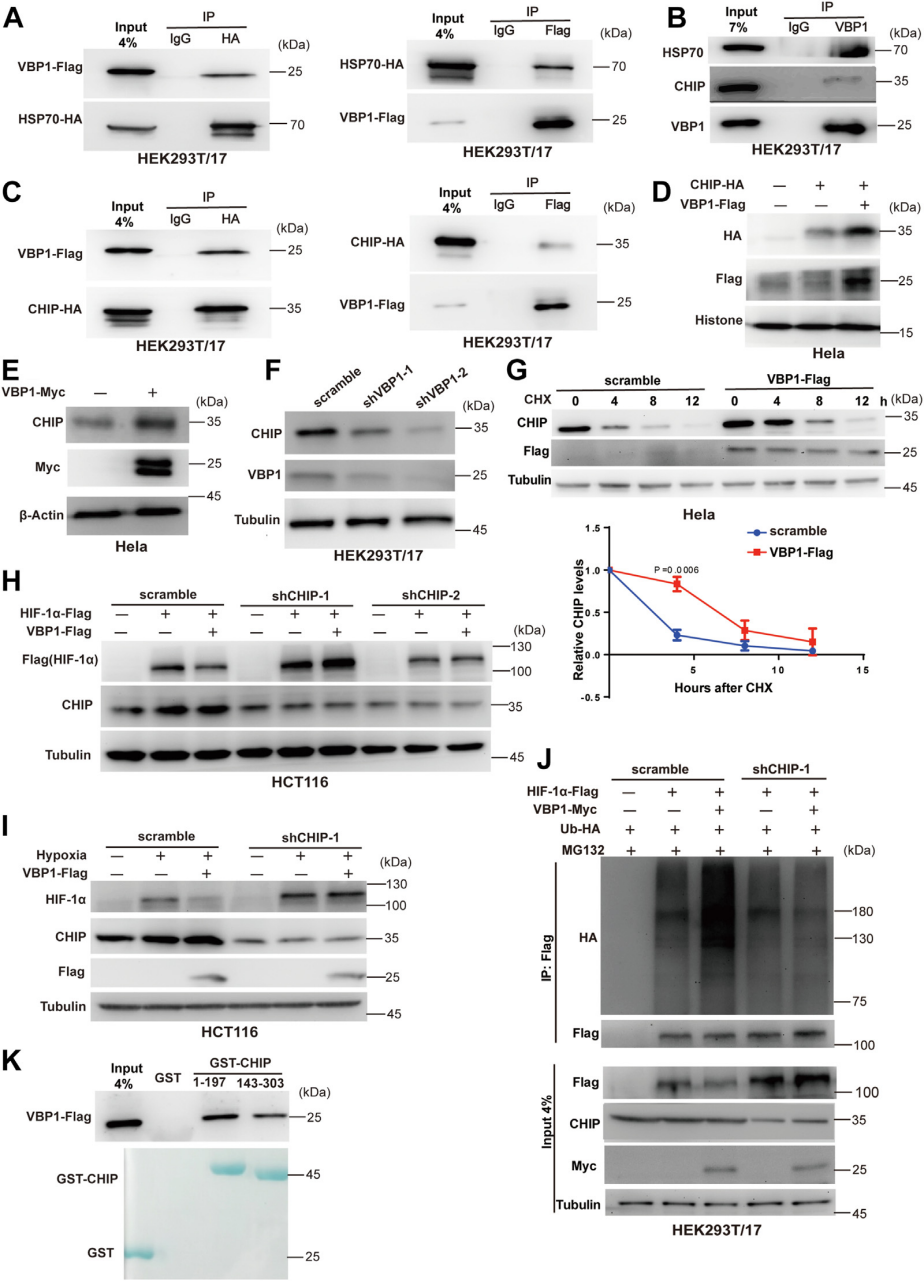
**CHIP is required for VBP1-mediated HIF-1α stabilization.***A*, Interaction between VBP1-Flag and HSP70-HA was analyzed in the HEK293T/17 cells by reciprocal Co-IP as indicated. *B*, Endogenous HSP70 and CHIP interact with VBP1 in the HEK293T/17 cells. Anti-VBP1 antibody was used for Co-IP. *C*, Interaction between VBP1-Flag and CHIP-HA was analyzed in the HEK293T/17 cells by reciprocal Co-IP as indicated. *D*, Overexpression of VBP1 increased the CHIP protein levels. HeLa cells were transfected with the indicated plasmids, and the proteins were detected by Western blotting analysis. *E*, VBP1 increased the endogenous CHIP protein levels. HeLa cells were transfected with VBP1-Myc, and the proteins were detected by Western blotting analysis. *F*, Western blotting analysis shows that CHIP protein levels were reduced in the VBP1-silenced HEK293T/17 cell lines. The HEK293T/17 cells were stably transfected with lentiviral pLKO plasmids cloned with specific shRNA targeting VBP1 or control scrambled shRNA. *G*, VBP1-Flag expression decreased CHIP protein half-life. HeLa cells with ectopic VBP1-Flag expression or control plasmids were treated with 50 μg/ml CHX for indicated time periods before being collected for western blotting analysis. The means and SDs from three independent experiments are shown (*lower panel*). *H*, Knockdown CHIP diminished the inhibitory effect on ectopic overexpressed HIF-1α by VBP1 in the HCT116 cells. Cells were transfected with the indicated plasmids, and the proteins were detected by Western blotting analysis. The bands of Flag-HIF-1α (∼110 Kd) and VBP1-Flag (∼25 Kd) were easily distinguished by molecular weight. *I*, Knockdown CHIP diminished the inhibitory effect on endogenous HIF-1α by VBP1 in cells. HCT116 cells transfected with VBP1-Flag or control plasmids were incubated under normoxia or hypoxia for 12 h, and the proteins were detected by Western blotting analysis. *J*, HEK293T/17 cells with or without silencing of CHIP expression were transfected with the indicated plasmids and treated with MG132 for 8 h. Cell lysates were immunoprecipitated with anti-Flag antibody. The immunoprecipitates and input were probed for indicated antibodies by immunoblotting. *K*, Mapping the interaction domain of CHIP with VBP1. GST pull-down assays were performed with the GST or GST fusion proteins containing different domains of the CHIP protein. The whole-cell lysates were prepared from HEK293T/17 cells expressing the Flag-tagged VBP1 protein. HIF-1, Hypoxia-inducible factor-1; HSP, heat shock protein; pVHL, von Hippel-Lindau protein; VBP1, pVHL binding protein 1.

**Supporting Figure S3:**
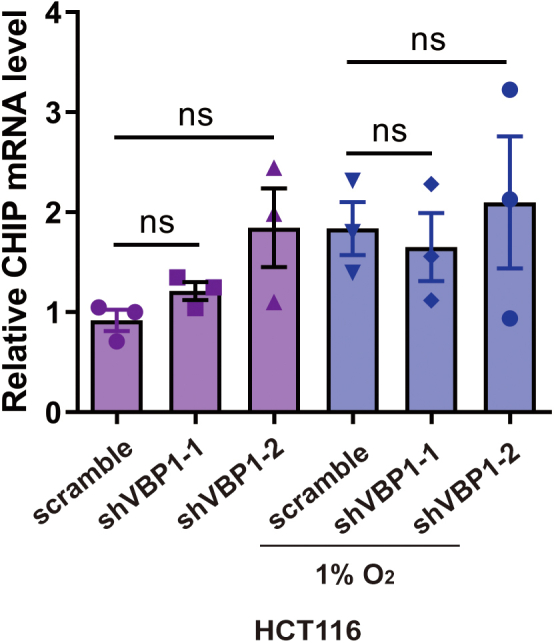


Next, we investigated the molecular mechanisms underlying the VBP1-induced degradation of HIF-1α. Toward this, we performed an IP pull-down assay followed by LC-MS/MS to identify the potential VBP1-binding proteins (data not shown). The MS results showed that HSP70 was a potential interacting protein of VBP1. We then validated the interaction between VBP1 and HSP70 using Co-IP assay in HEK293T/17 cells transfected with the Flag-tagged VBP1 and HA-tagged HSP70. Co-IP results demonstrated that VBP1 and HSP70 were co-precipitated by the corresponding antibodies suggesting the interaction between VBP1 and HSP70 (Fig. 6*A*). Furthermore, Co-IP results showed that VBP1 interacted with the endogenous HSP70 (Fig. 6*B*). Previous studies have shown that HSP70 promotes the degradation of HIF-1α by recruiting CHIP, a ubiquitin ligase protein (35). Therefore, we examined the interaction between VBP1 and CHIP. Co-IP assay results showed that CHIP interacted with VBP1(Fig. 6, *B* and *C*). HeLa cells co-transfected with plasmids expressing Flag-tagged VBP1 and HA-tagged CHIP showed increased CHIP protein levels (Fig. 6*D*). Furthermore, overexpression of VBP1 increased endogenous CHIP levels (Fig. 6*E*). However, VBP1 overexpression did not affect the levels of CHIP mRNA (Fig. S3). Conversely, the knockdown of VBP1 reduced CHIP protein levels (Fig. 6*F*). To investigate whether VBP1 affects protein stability of CHIP, the protein half-life of CHIP was analyzed. HeLa cells with Flag-tagged VBP1 expression or their control cells were treated with protein synthesis inhibitor cyclohexamide (CHX) for different time periods. Compared with control cells transduced with the empty vector, cells transduced with Flag-tagged VBP1 exhibited an increased half-life of CHIP protein (Fig. 6*G*), suggesting that VBP1 stabilizes CHIP protein. We, therefore, hypothesized that CHIP modulated the function of VBP1. In agreement with our hypothesis, CHIP knockdown reduced VBP1-induced downregulation of HIF-1α in the HCT116 cells (Fig. 6, *H* and *I*). Also, although ectopic expression of VBP1 increased the ubiquitination of HIF-1α in the control cells, we did not detect an obvious difference in the ubiquitination of HIF-1α in the CHIP silenced cells (Fig. 6*J*). We then mapped the domains of CHIP that were involved in CHIP/VBP1 binding by performing GST pull-down assay with the GST-tagged fragments of CHIP protein and protein lysates of HEK293T/17 cells transfected with the Flag-VBP1 plasmids. GST pull-down assays demonstrated that both GST-CHIP (1–197) and GST-CHIP (143–303) interacted with VBP1 (Fig. 6*K*). Taken together, these data demonstrated that CHIP was required for VBP1-mediated degradation of HIF-1α.

We then analyzed if CHIP was sufficient to mediate HIF-1α degradation in the absence of VBP1. CHIP significantly reduced HIF-1α protein levels in the HCT116 cells transfected with scrambled control shRNA, but VBP1 knockdown prevented CHIP-induced HIF-1α degradation (Fig. S4*A*). We further evaluated the effects of HSP70 on the HIF-1α stability in VBP1-deficient cells. However, the knockdown of VBP1 did not affect HSP70-mediated HIF-1α degradation (Fig. S4*B*). This suggested that VBP1 was not involved in HSP70-induced HIF-1α degradation. Taken together, our study demonstrated that VBP1 and CHIP co-operatively regulated HIF-1α protein degradation.

### Low VBP1 expression is associated with poor survival outcomes in ccRCC

**Figure 7:**
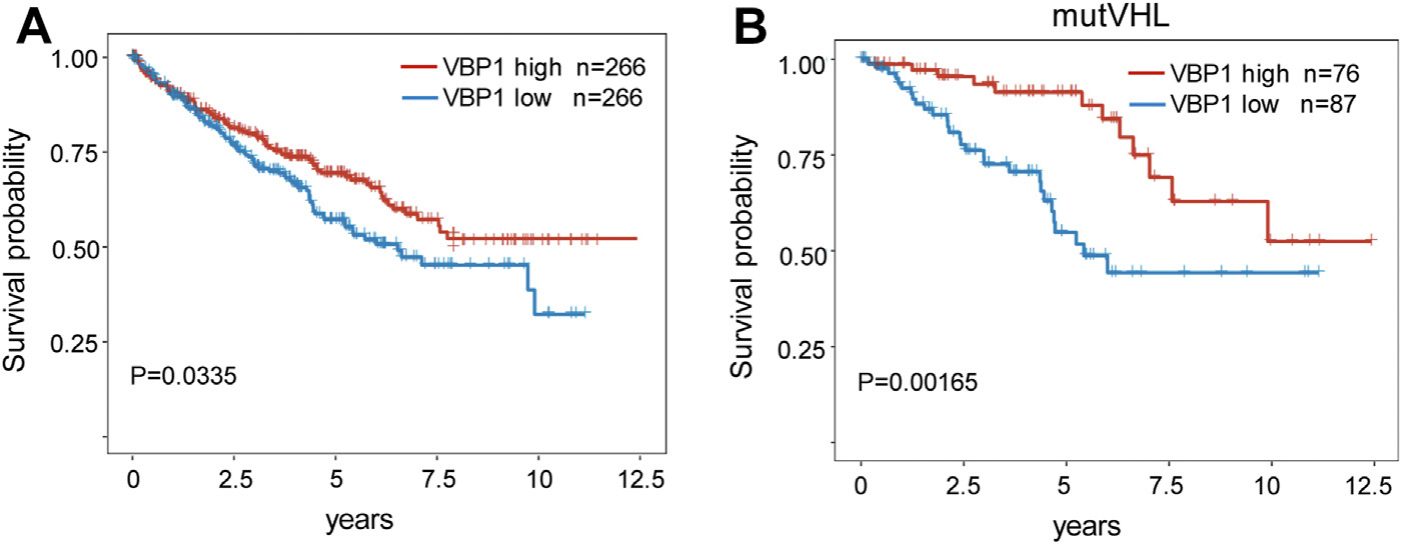
**VBP1 expression is associated with the overall survival of ccRCC patients.***A*, Kaplan-Meier survival curves revealed that patients with low levels of VBP1 (bottom 50%, TPM < 5.94633) had significantly lower overall survival in the ccRCC, n = 532. *B*, Patients with mutated VHL with low levels of VBP1 (TPM < 5.94633) had significantly lower overall survival in the ccRCC, n = 163. RNA-seq expression profiles of *VBP1* gene and corresponding clinical information for ccRCC and ccRCC *VHL* mutant patients were obtained from TCGA database. All data were standard TPM data and the statistical analyses were performed using R software. ccRCC, clear cell renal carcinoma; OS, overall survival; VBP1, pVHL binding protein 1.

The HIF signaling pathway is constitutively activated in the clear cell renal carcinoma (ccRCC) cells because of the loss of pVHL. In ccRCC, HIF-1α had been proposed as a tumor suppressor, but recent studies revealed an oncogenic role of HIF-1 as an independent indicator of poor outcome (7, 36, 37, 38). Therefore, we assessed the effects of VBP1 on the overall survival (OS) of patients with ccRCC. Kaplan-Meier survival analysis of patients with ccRCC from the TCGA database showed that patients with low VBP1 expression were significantly associated with poorer OS (Fig. 7*A*). Furthermore, among patients with mutated pVHL (n = 163), those with low VBP1 expression (n = 87) showed worse OS than those with high VBP1 expression (Fig. 7*B*), indicating a pVHL-independent role of VBP1 in the ccRCC patients.

## Discussion

Recent studies have reported that VBP1 performs tumor suppressor functions through multiple mechanisms such as HIF-1α degradation in a pVHL-dependent manner and as a co-chaperone in the correct folding of newly synthesized pVHL (23, 30). In the current study, we uncovered a novel mechanism by which VBP1 regulated pVHL-independent HIF-1α degradation. We identified CHIP as a key factor responsible for VBP1-induced HIF-1α degradation. Furthermore, genetic experiments in zebrafish demonstrated a key role for VBP1 in HSC formation under hypoxic conditions. We also confirmed for the first time that VBP1 modulated HIF-1α stability and degradation *in vivo*. Finally, we demonstrated that patients with ccRCC having low VBP1 expression levels were associated with worse OS than those with high VBP1 levels.

A previous *in vitro* study reported that VBP1 stabilized HIF-1α (30). However, the *in vivo* relationship between VBP1 and HIF signaling has not been well established. In this study, we performed *in vivo* experiments using the zebrafish embryos to demonstrate the regulatory role of VBP1 in HIF-1 signaling. In contrast to the *in vitro* cell culture studies, the whole zebrafish study allowed us to analyze the biological functional effects of VBP1 by regulating the HIF-1 signaling pathway. To this end, we generated a HIF reporter zebrafish named *Tg(hre-sv40mp:GFP)* and *vbp1* mutants to analyze HIF protein levels and transcriptional activity. When subjecting the transgenic embryos to hypoxia-induced by physical or genetic conditions, we observed strong expression of GFP. These transgenic embryos are suitable for identifying novel chemicals or genes that affect Hif activity. Our previous study showed that *vbp1* mutants were embryonic lethal by 10 dpf (29). In this study, we demonstrated that Vbp1 overexpression reduced the GFP fluorescence intensity of *Tg(hre-sv40mp:GFP)* embryos in the *vhl* mutant background. Furthermore, Hif activity was significantly high in the *vbp1* mutants. Therefore, we demonstrated that Vbp1 inhibited Hif signaling in zebrafish.

The stability of HIF proteins is regulated by both pVHL-dependent and -independent mechanisms (19, 39, 40). Our data demonstrated that VBP1 was a key modulator of HIF-1α but not HIF-2α. Although VBP1 increased pVHL-dependent HIF-1α degradation (30), this study clearly showed that HIF-1α underwent pVHL-independent degradation *via* VBP1. We have provided multiple lines of evidence to show that VBP1 modulated HIF-1α protein stability independent of pVHL: (1) VBP1 silencing in pVHL-deficient RCC4 cells increased the levels of HIF-1α protein, whereas overexpression of VBP1 significantly decreased HIF-1α protein levels; (2) VBP1 interacted with the hydroxylation-mutant HIF-1α (P402A/P564A) and reduced the protein levels and the transcriptional activity of HIF-1α (P402A/P564A); (3) Vbp1 downregulated Hif signaling pathway in the homozygous *vhl* mutant zebrafish; (4) low levels of VBP1 were associated with poorer survival of pVHL mutated ccRCC patients. Thus, our data strongly demonstrated that VBP1 regulated HIF-1α stability and degradation through a pVHL-independent mechanism.

However, our findings contrast with those of Kim's study (30), which reported no physical interaction between VBP1 and HIF-1α in human colorectal carcinoma HCT116 cells. On the other hand, our study confirms that HIF-1α can bind to VBP1 in multiple cell types, including HEK293T/17 and HeLa cells. Further investigation is necessary to determine whether the inconsistent results were due to variations in cell lines. Additionally, the earlier study suggested that VBP1 facilitated pVHL degradation, leading to HIF-1α stabilization. We argue against the use of tubulin as an internal control, as it has been demonstrated that VBP1 is essential for tubulin stability (25). Therefore, we selected GAPDH as an internal control, and our results strongly support a pVHL-independent regulatory mechanism for VBP1.

VBP1 is an essential co-chaperone for the folding of tubulin during cytoskeleton assembly (25). Furthermore, VBP1 is required for preventing the aggregation of functional proteins such as pVHL, HIV integrase, and others by facilitating their degradation (25, 27, 28). Our recent study has confirmed that VBP1 promoted the degradation of TCF7L1 and TCF7L2 (29). Importantly, these studies have confirmed that pVHL and VBP1 cooperate to control the target protein degradation. In this study, we revealed for the first time that VBP1 degraded HIF-1α independent of pVHL. Also, we provided evidence that VBP1 promoted the degradation of HIF-1α in a folding-independent manner, indicating that the chaperone function of the VBP1 was not involved in HIF-1α stability.

One critical question raised is how VBP1 regulates HIF-1α in a pVHL-independent manner. We identified CHIP as a key player in VBP1-mediated HIF-1α degradation. CHIP is a highly conserved ubiquitin E3 ligase that participates in the ubiquitination of multiple substrates such as c-Myc, p53, EGFR, HIF-1α, and others (41, 42, 43). Interestingly, a previous study showed that CHIP regulated the degradation of HIF-1α but not HIF-2α during prolonged hypoxia (35). In this study, we have demonstrated that VBP1 physically interacted with CHIP, and CHIP expression levels were positively regulated by VBP1. Notably, CHIP knockdown abrogated HIF-1α degradation by VBP1. CHIP has been reported to have both oncogenic and tumor-suppressing functions (17). Our finding suggested that CHIP potentially played a tumor-suppressor role through HIF-1α degradation. Nevertheless, further studies are necessary to gain a better understanding of the molecular interplay between VBP1 and CHIP.

In conclusion, our present studies for the first time disclose a novel mechanism of VBP1 in the modulation of HIF-1α stabilization through the regulation of CHIP expression. A better understanding of the roles of VBP1 in CHIP-mediated HIF-1α stability may provide further insights into their roles in human diseases including cancers and contribute to the development of drugs targeting HIF-1α signaling.

## Experimental procedures

### Animals

Zebrafish (*Danio rerio*) were maintained on a 14 h/10 h light/dark cycle at 28 °C and fed twice daily. The *vbp1* mutants were constructed as previously described (29). The *vhl* mutants were obtained as gifts from the laboratory of Wuhan Xiao (Institute of Hydrobiology, Chinese Academy of Sciences). The fertilized zebrafish eggs were staged and maintained according to the standard methods described previously (44). The embryo medium was supplemented with 0.003% (w/v) 2-phenylthiourea to prevent pigmentation. The experimental protocols were approved by the Ethical Committee of Experimental Animal Care, Ocean University of China.

### Generation of transgenic zebrafish

The HRE-SV40mp promoter was constructed with a 68-bp PCR-amplified fragment from the hypoxia-responsive element (HRE) of the human ENO1 gene (5′-AGGGCCGGACGTGGGGCCCCAGAGCGACGCTGAGTGCGTGC-GGGACTCGGAGTACGTGACGGAGCCCC-3′) and the SV40 minimal promoter (SV40mp) as previously described for the construction of a luciferase reporter vector (45). In the present study, the HRE-SV40mp promoter was amplified by PCR from the p2.1 (pAGL2A) plasmid and cloned into pT2AL200R150G (46). The reporter plasmid DNA and the Tol2 transposase mRNA were injected into one-cell stage zebrafish embryos. The transgenic fish expressing GFP under hypoxia were outcrossed with the wild-type fish to produce the founder line. The transgenic fish were outcrossed with the wild-type fish to produce the *Tg (hre-sv40mp: GFP)* transgenic line.

### Plasmid constructs

HA-HIF1alpha P402A/P564A-pBabe-puro and HA-HIF2alpha-P405A/P531A-pBabe-puro were gifts from William Kaelin (Addgene plasmid #19005; #19006) (47). The plasmids pCDH-Flag-HIF-1α, Pb-3 × Flag-HIF-1α, pCS2 -VBP1-Myc, pCS2-Flag-VBP1, pBobi-Flag-VBP1, pBobi-Flag-VHL, pCDNA3.1 to 3 × HA-CHIP, pCDNA3.1 to 3 × HA-HSP70, pCS2-Flag-ΔVBP1, and pCDNA3.1-PFDN5-2 × FLAG, were generated by PCR subcloning as previous described (29). The following truncation mutant plasmids were generated to analyze the motifs or domains in HIF-1α, VBP1, CHIP, and HSP70: pGEX-4T-GST-ΔHIF-1α(1–80aa), pGEX-4T-GST-ΔHIF-1α(81–200aa), pGEX-4T-GST-ΔHIF-1α(201–329aa), pGEX-4T-GST-ΔHIF-1α(330–427aa), pGEX-4T-GST-ΔHIF-1α(428–531aa), pGEX-2T-GST-ΔHIF-1α(532–826aa), pGEX-2T-GST-ΔCHIP(1–197aa), pGEX-4T-GST-ΔCHIP(143–303aa), pGEX-2T-GST-ΔHSP70(1–441aa), and pGEX-4T-GST-ΔHSP70(442–641aa). The pLKO.1-GFP-CHIP-sh1 and pLKO.1-GFP-CHIP-sh2 plasmids were used to generate stable cell lines. The restriction sites as well as the Kozak sequence were also cloned into the plasmids with the primers. All the primers used in this study are shown in Table S1.

### Cell lines and transfections

HEK293T/17, HeLa, HCT116, and U2OS cell lines were purchased from ATCC. The RCC4 cell line was gifted by Wuhan Xiao (Institute of Hydrobiology, Chinese Academy of Sciences) and cultured in high-glucose DMEM medium with 10% FBS, 100 units/ml penicillin, and 100 mg/ml streptomycin sulfate at 37 °C and 5% CO_2_. The cell lines were authenticated using short tandem repeat profiling by the ShCellBank (Shanghai, China). *Mycoplasma* contamination in the culture cells was assessed using the EZ-PCR Mycoplasmas Detection Kit (BI, Kibbutz Beit-Haemek, Israel) every 3 months. The cells were seeded into 35-mm tissue culture dishes and transfected with 200 ng of plasmid DNA using PEI according to the manufacturer’s instructions.

### mRNA synthesis and microinjection

Capped Vbp1 or Hif-1α DA mRNA synthesis was carried out using linearized plasmid DNA as a template and a mMessage mMachine mRNA kit (46). The synthesized mRNA was diluted with phenol red dye to 10 ng/μl and approximately 1 nl were microinjected into 1 to 2 cell stage zebrafish embryos. After injection, the embryos were raised in ERS and maintained at 28.5 °C.

### Hypoxia experiments

A humidified modular incubation chamber was used to generate hypoxia in the cultured cells. The cell culture was exposed in the hypoxia chamber to a gas mixture with 1% oxygen, 5% carbon dioxide, and 94% nitrogen, which was injected through one end of the tube. The other end of the tube was connected to the exhaust. After 10 min, ventilation was stopped and the two ends of the tube were clamped quickly, and the cell culture dish was placed in the 37 °C incubator. After 24 or 48 h, the hypoxic chamber was removed, the cover was carefully opened, and the cells were harvested for subsequent experiments.

### Antibodies and reagents

The following primary antibodies were used in this study: HIF-1α (#610959, BD Biosciences); HIF-2α (#NB100-122, Novus Biologicals); Tubulin (#200608, ZEN BIO); VBP1 (#sc-390465), VHL (#sc-135657), MYC (#sc-40), CHIP (#sc-133066), HSP70(#sc-66048) were all purchased from Santa Cruz Biotechnology; HA (#3724; Cell Signaling Technology); GAPDH (#D110016, BBI life sciences); histone H3 (#P30266; Abmart); FLAG M2 (#F1804; Sigma-Aldrich) and β-actin (abs137975; Absin Biosciences). MG132 (HY-13259) and BAY 87-2243 were purchased from MedChemExpress.

### Immunoblots, Co-immunoprecipitation (IP), and GST pull-down assay

For immunoblots, 1 × 10^6^ cells were seeded in 35-mm dish and the cells were harvested at 24 h post-transfection; then the cells were lysed with RIPA buffer containing 150 mM NaCl, 50 mM Tris, 0.5% NP-40, 1 mM EDTA, 0.1% SDS, PH 7.5, with protease inhibitors (Roche) at 4 °C for 30 min followed by centrifugation at 12,000*g* for 10 min at 4 °C to remove the debris. Protein samples were mixed with 5 × loading buffer and then separated by SDS/PAGE. After transferring to PVDF membranes and blocking with 5% nonfat milk, then incubated with primary antibodies overnight at 4 °C. Primary antibodies were detected with HRP-conjugated secondary antibody (Beyotime Biotechnology).

For Co-IP experiments, transiently transfected cells in 100-mm dishes were harvested by scraping directly into lysis buffer at 48 h post-transfection. The cells were then lysed in IP lysis buffer containing 50 mM Tris, pH 7.5, 150 mM NaCl, 1 mM EDTA, 10% glycerol, 1% Triton X-100, and protease and phosphatase inhibitors. Cleared cell lysates were incubated with the desired antibody overnight at 4 °C and immunoprecipitated by incubating with Protein-A/G beads at 4 °C for 6 h. The beads were washed with IP lysis buffer four times and using SDS loading buffer for immunoblot analysis.

For the GST pulldown assay, *Escherichia coli* BL21 competent cells were transformed as described previously (29). Briefly, the competent cells were transformed with GST or GST-VBP1 and then induced with 0.1 mM isopropyl b-D-thiogalactopyranoside at 37 °C for 5 h. The cells were collected by centrifugation, 6000 rpm for 8 min and lysed in lysis buffer (50 mM Tris, pH 7.5, 150 mM NaCl, 1 mM EDTA, 10% glycerol, and 1% Triton X-100) on ice, sonicated for 30 min. Glutathione Sepharose 4B (GE Healthcare) was performed and mixed with the GST or GST-VBP1 extraction overnight on a rotator at 4 °C. These mixtures were centrifuged, and the precipitates were diluted with an equal amount of indicated cell lysates of HEK293T/17 and rotated for 2 h at 4 °C. After extensive washing of the beads with the lysis buffer, the precipitated proteins were eluted using SDS/PAGE. The bound proteins were detected by indicated antibodies or Coomassie Blue staining.

For the ubiquitylation assay, we treated the protein with hot lysis buffer based on the protocol previously described (29). Briefly, HEK293T/17 cells were cotransfected with HA-Ub, Flag-HIF-1α, and VBP1-Myc, along with an empty vector. The cells were then treated with 20 mM MG132 for 8 h before harvesting. We lysed the protein in “hot SDS” lysis buffer (2% SDS, 150 mM NaCl, and 10 mM Tris-HCl, pH 8.0) with 2 mM N-ethylmaleimide and protease inhibitors, and lysates were boiled for 10 min and then diluted with nine volumes of dilution buffer (10 mM Tris-HCl, pH 8.0, 150 mM NaCl, 2 mM EDTA, and 1% Triton). The samples were incubated at 4 °C for 30 to 60 min with rotation and precleared by centrifugation at 12,000 3*g* for 5 min before performing immunoprecipitation.

### Generation of stable cell lines

The pLKO.1-GFP-CHIP-sh1 and pLKO.1-GFP-CHIP-sh2 plasmids were transfected with the psPAX2 and pMD2 plasmids to generate the lentiviruses in the HEK293T/17 cells. Briefly, 2 × 10^6^ HEK293T/17 cells were seeded in a 60-mm petri-dish with 4 ml of DMEM medium containing 10% FBS without any antibiotics. Next day, psPAX2, pMD2.G, and the lentivirus vector pLKO.1-GFP containing the puromycin resistance gene, and the shRNA sequences (pLKO.1-GFP-CHIP-sh1 or pLKO.1-GFP-CHIP-sh2) were co-transfected into the packaging cell line using Lipofectamine 2000 according to the manufacturer’s instructions. After 18 h, the transfection medium was replaced with fresh DMEM medium with 10% FBS and further incubated for 72 h. Then, the medium containing the lentivirus was harvested and concentrated. The target cells were infected with the lentivirus in 8 mg/ml polybrene. After 72 h, we added 1.0 mg/ml puromycin to the infected culture plates. The medium was changed every 2 or 3 days. The stably transformed cells were harvested when they became confluent and were cryo-preserved as a polyclonal line.

### Dual luciferase reporter assay

To examine the regulation of HIF by VBP1 *in vitro*, the HEK293T/17 cells were co-transfected with vectors carrying VBP1, HIF, and the ENO1 promotor-luciferase reporter or VEGF promoter-luciferase reporter. The transfection efficiency was estimated by co-transfecting the renilla luciferase reporter plasmid, pRLSV40, as previously reported (44). Luciferase activity was measured in the transfected cells using the Dual-Luciferase Assay System (Promega). The relative firefly luciferase activity was estimated by normalizing to the Renilla luciferase activity and was expressed as the fold increase over the control group.

### Whole-mount *in situ* hybridization

Whole-mount *in situ* hybridization was performed as described previously (48). Briefly, the embryos were fixed overnight with 4% (w/v) paraformaldehyde in PBS at 4 °C. After washing, the embryos were stored in methanol at −20 °C until use. The information for zebrafish *cmyb/runx1* probes was listed in Table S1. The labeled embryos were mounted in glycerol or 5% methylcellulose, respectively, and were photographed using a Nikon SMZ1500 microscope.

### RNA extraction and real-time quantitative PCR

Total RNA was extracted using the RNAiso plus reagent (Takara Bio). Then, cDNA was prepared from 2 μg total RNA using the M-MLV reverse transcriptase kit (Promega) according to the manufacturer’s protocol. Subsequently, q-PCR was performed with specific primers and iTaq SYBR GREEN Supermix in a BioRad iCycler (Bio-Rad). The primers were shown in Table S1. The relative mRNA levels of each target gene were calculated by normalizing them to the β-actin mRNA levels based on the 2^−ΔΔCT^ method. In all RT-qPCR experiments, each unknown sample was estimated in duplicate.

### Analysis with online database

The analysis of ccRCC was conducted through multiple integrative web-based platforms in bioinformatics. The expression patterns of VBP1 in normal and patients with ccRCC from TCGA (The Cancer Genome Atlas) were studied using the online website HOME for Researchers (https://www.home-for-researchers.com/static/index.html#/tcga). The RNA-Seq expression profiles and corresponding clinical information for VBP1 were obtained from the TCGA dataset (https://www.cancer.gov/about-nci/organization/ccg/research/structural-genomics/tcga). To evaluate the impact of VBP1 on survival, the Kaplan–Meier plotter (http://www.kmplot.com/analysis/) was utilized, which included 532 samples from patients with ccRCC and 163 samples from patients with mutated VHL. All data were standardized as Transcripts per million, and R software was used for statistical analysis.

### Statistical analysis

GraphPad Prism 8.0 software (GraphPad Prism software) was used for statistical analysis. The data are represented as means ± SD. The statistical differences between groups were analyzed by one-way or two-way ANOVA or the Student’s *t* test. *P < 0.05* was considered statistically significant. All the experiments were performed at least in triplicates for every condition.

## Data availability

All data are contained within the article.

## Supporting information

This article contains supporting information.

## Conflict of interest

The authors declare that they have no conflicts of interest with the contents of this article.

## Figures and Tables

**Supporting Figure S4:**