# Supplementary material for: VBP1 negatively regulates CHIP and selectively inhibits the activity of hypoxia-inducible factor (HIF)-1α but not HIF-2α
Source: J Biol Chem. 2023 May 16;299(6):104829. doi: 10.1016/j.jbc.2023.104829 (PMC10318525; doi:10.1016/j.jbc.2023.104829)
Supplement: Supporting information [file mmc1.docx]

**Supporting information text**

**Figure S1. *Tg(hre-sv40mp:GFP)* zebrafish as a live reporter of hypoxia signaling pathway**.

(A) *Tg(hre-sv40mp:GFP)* embryos at 48 hpf were treated for 24 h with 0.1% DMSO or 100 μM hydroxylase inhibitor CoCl_2._ The CoCl_2_-treated embryos showed an increase in the GFP expression. (B) Robust expression of GFP in the *vhl^-/-^* larvae at 5, 9, 12 dpf compared with sibling larvae. (C) Effect of HIF-1α inhibition in *Tg(hre-sv40mp:GFP)* zebrafish. Transgenic embryos at 24 hpf were exposed to 200 nM BAY 87-2243 for 24 h under hypoxia conditions. 200 nM BAY 87-2243 inhibited GFP fluorescence compared with DMSO control group. Scale bar, 500 μm.

**Figure S2. Knockdown of VBP1 has no effect on HIF-1α mRNA levels.**

HEK293T/17 cells stably transfected with lentiviral pLKO shRNA targeting VBP1 or nontargeting control (scramble) and the mRNA was analyzed by qRT-PCR.

**Figure S3. Knockdown of VBP1 has no effect on CHIP mRNA levels.**

HCT116 cells stably transfected with lentiviral pLKO shRNA targeting VBP1 or nontargeting control (scramble) were exposed to either 20% or 1% O_2_ for 6 h. Afterward, the mRNA was analyzed by qRT-PCR.

**Figure S4. Knockdown VBP1 prevented CHIP-induced HIF-1α degradation but has no effect on HSP70-mediated HIF-1α degradation.**

(A) VBP1 knockdown prevented the decrease of CHIP-induced HIF-1α protein. HCT116 cells were transfected with the indicated plasmids, and the proteins were detected by Western blotting analysis. (B) HCT116 cells were transfected with the indicated plasmids, and the proteins were detected by Western blotting analysis. (-), the cells transfected with the empty vector control; (+), the cells transfected with the indicated vector.

**Table S1 The primer sequences**
