# Supplementary material for: VBP1 negatively regulates CHIP and selectively inhibits the activity of hypoxia-inducible factor (HIF)-1α but not HIF-2α
Source: J Biol Chem. 2023 May 16;299(6):104829. doi: 10.1016/j.jbc.2023.104829 (PMC10318525; doi:10.1016/j.jbc.2023.104829)
Supplement: Supporting Table S1 [file mmc2.docx]

| **Primer Name** | **Sequence** |
| --- | --- |
| HIF-1α-F | gatgacgatgacaagggatccATGGAGGGCGCCGGCGGCGCGAA |
| HIF-1α-R | caagaaagctgggtcttaattaaTCAGTTAACTTGATCCAAAGCTCTG |
| VBP1-F | CGCCTTGTTCTTCTCCTT |
| VBP1-R | ACAACACTTGCACAGCATT |
| CHIP-F | CGGGATCCGCCACCATGAAGGGCAAGGAGGAG |
| CHIP-R | CGGAATTCGTAGTCCTCCACCCAGCCAT |
| HSP70-F | CGGGATCCGCCACCATGGCCAAAGCCGCGGCGAT |
| HSP70-R | CG GAATTC ATCTACCTCCTCAATGGTGG |
| HIF-1α(1-80aa)-F | GGGGTACCGCCACCATGGAGGGCGCCGGCGGCGC |
| HIF-1α(1-80aa)-R | CGGGATCCGAATATCCAAATCACCAGCATCC |
| HIF-1α(81-200aa)-F | GGGGTACCGCCACCATGGAAGATGACATGAAAGCAC |
| HIF-1α(81-200aa)-R | CGGGATCCGTACGTGAATGTGGCCTGTGC |
| HIF-1α201-329aa)-F | GGGGTACCGCCACCATGTATGATACCAACAGTAACC |
| HIF-1α(201-329aa)-R | CGGGATCCGATTCTTGGTGTTATATATG |
| HIF-1α(330-427aa)-F | GGGGTACCGCCACCATGTCTCAACCACAGTGCATTG |
| HIF-1α(330-427aa)-R | CGGGATCCGTTCCTCAAGTTGCTGGTCATCAG |
| HIF-1α(428-531aa)-F | GGGGATCCGCCACCATGGTACCATTATATAATGATG |
| HIF-1α(428-531aa)-R | AAGGAAAAAAGCGGCCGCGTGAATTCATTGACCATATCAC |
| HIF-1α(532-826aa)-F | GGGGTACCGCCACCATGAAGTTGGAATTGGTAGAA |
| HIF-1α(532-826aa)-R | CGGGATCCGGTTAACTTGATCCAAAGCTC |
| GST-HIF-1α(532-826aa)-F | CGGGATCCAAGTTGGAATTGGTAGAAAAC |
| GST-HIF-1α(532-826aa)-R | CGGAATTCTCAGTTAACTTGATCCAAAGCTC |
| ΔPFDN3(BamH1)-F | CGGGATCCATGCACCTGGGGATTCCTGAGGC |
| ΔPFDN3(EcoR1)-R | CGGAATTCTTATTCTGTGGTAGTAAATTGATC |
| PFDN5(BamH1)-F | CGGGATCCATGGCGCAGTCTATTAACAT |
| PFDN5(EcoR1)-R | GGAATTCGGCCTTAGCAGTAGCCTGAG |
| GST-CHIP(1-197aa)-F | CGGGATCCAAGGGCAAGGAGGAGAAGGAG |
| GST-CHIP(1-197aa)-R | GGAATTCTCACTGCTGGGCCCGGACGTGG |
| CHIP SH1 | ACCACGAGGGTGATGAGGA |
| CHIP SH2 | GCAAGGACATCGAGGAGCA |
| PFDN1-F | CGGGATCCATGGCCGCCCCCGTGGATCT |
| PFDN1-R | GGAATTCCTGGGCCCTTCGTGCCATC |
| Cited2-F | GTTCCGAGACAGTATCGCTAAG |
| Cited2-R | ATCAAGACCTCCTCGTCAATAA |
| Pai1-F | ATTCCAAGGTTCTCCATGGA |
| Pai1-R | GGTTCCTCAGTAGTAATGCG |
| il11a-F | CCGGGTGTTTAGTACAGAGATT |
| il11a-R | CATGGAGCTGAGAAAGAGTAGG |
| β-Actin-F | AGGTCATCACCATTGGCAAT |
| β-Actin-R | GATGTCGACGTCACACTTCAT |
| Redd1-F | TGGACTCTGACTCCGACAACC |
| Redd1-R | ACCACTTCTTTACACAACGCCTC |
| Gadd34-F | GAAGAGCAGTGGGAAGAAGG |
| Gadd34-R | CTGAACTCTCCTCCTGAAACG |
| Epo-F | AGGAGGCAGGATATGGACTATTAC |
| Epo-R | ACAGTTGGAGGTGCTTGAGG |
| PGK-F | AAGTCGGTAGTCCTTATGAGC |
| PGK-R | CACATGAAAGCGGAGGTTCT |
| ACTIN-F | AGAGCTACGAGCTGCCTGAC |
| ACTIN-R | AGCACTGTGTTGGCGTACAG |
| CHIP-F | CATATCTCACCAGGCTCATTGC |
| CHIP-R | TATCTGCCATGTATTTATCGTGCTTG |
| HSP70-F | ATGCGCTCGAGTCCTACGCCTT |
| HSP70-R | GCTGATCTTGCCCTTGAGACCCTC |
| cmyb-probe F | AGGGACAACAGGCACTACCA |
| cmyb-probe R | TGTTGGGAAGCATCAGCACA |
| runx1-probe F | GACAGCCAATAGCACGCCGGCA |
| runx1-probe R | AGTGTTTTCAATGGAGTTTCT |
